# Maternal Obesity, Non-Respiratory Sleep Symptoms, Prescribed Nutritional Supplements and Routine Hematological Indices During Pregnancy: A Single-Center Prospective Pilot Study

**DOI:** 10.3390/nu18132186

**Published:** 2026-07-05

**Authors:** Verónica López-García, Sergio Galarreta-Aperte, Piedad Gómez-Torres, Beatriz García-López, Natalia García-Ruiz, Francisco de Asís Membrive-Jiménez, María José Membrive-Jiménez, David Peña-Otero

**Affiliations:** 1 Multidisciplinary Sleep and Ventilation Unit J. Terán Santos, University Hospital of Burgos, 09001 Burgos, Spain; vlopezg@saludcastillayleon.es (V.L.-G.); ngarciaru@saludcastillayleon.es (N.G.-R.); 2 Department of Nursing, Faculty of Health Sciences of Ceuta, University of Granada, 51001 Ceuta, Spain; mjmembrive@ugr.es; 3 Area of Human Anatomy and Embryology, Health Sciences, University of Burgos, 09001 Burgos, Spain; bglopez@ubu.es; 4 Department of Cell Biology, Faculty of Sciences, University of Granada, 18071 Granada, Spain; franmembri@correo.ugr.es; 5 Institute for Biosanitary Research (ibs.GRANADA), 18012 Granada, Spain; 6 Nursing Research Unit, Marqués de Valdecilla Research Institute (IDIVAL), 39008 Santander, Spain; david.penha.otero@gmail.com; 7 Marqués de Valdecilla University Hospital, Cantabrian Health Service, 39008 Santander, Spain

**Keywords:** pregnancy, maternal obesity, insomnia, sleep fragmentation, daytime sleepiness, prescribed nutritional supplements, hematological indices, pilot study

## Abstract

**Background/Objectives:** Pregnant women with obesity may experience a substantial burden of sleep disruption and obstetric risk, but prospective data integrating non-respiratory sleep symptoms, prescribed nutritional supplement exposure, and routinely collected hematological measures are limited. This single-center prospective pilot study aimed to describe longitudinal changes in anthropometry, non-respiratory sleep symptoms, prescribed nutritional supplement exposure, routine hematological and blood-count-derived inflammatory indices, and obstetric and neonatal outcomes, and to document study-accrual and data completeness metrics for a future controlled cohort. **Methods:** Seventeen adult women with singleton pregnancies and first-trimester body mass index (BMI) ≥ 30 kg/m^2^ reached the sleep unit, and all consented to participate. Protocol-based assessments were performed in the first and third trimesters, whereas routine laboratory and treatment data were extracted from medical records. Nutritional exposure was defined solely by prescription status in routine records; doses, formulations, adherence, dietary intake, and serum micronutrient markers were unavailable. Analyses were endpoint-specific complete-case analyses. **Results:** Study accrual at the sleep unit occurred from 27 July 2023 to 14 March 2024 (17 participants over 7.6 months; approximately 2.2 participants/month). The number informed in Obstetrics and the number not reaching the sleep unit were not recorded. Paired anthropometry was available for 15/17 participants, paired Epworth and Insomnia Severity Index scores for 14/17, paired restless-legs scores for 13/17, and delivery outcomes for 16/17. Median paired changes were 6.0 kg (IQR 1.4–7.9) for weight and 2.3 kg/m^2^ (IQR 0.5–3.1) for BMI. Hemoglobin decreased, and selected blood-count-derived indices varied across routine trimester measurements. Sleep scores showed no clear longitudinal change, although nocturnal awakenings and subclinical insomnia were frequent. Type III polygraphy was attempted in all 17 participants at T1 and was valid in 16; one participant declined a repeat study after a technically invalid recording. Polygraphy was valid in 15 participants at T3; a respiratory event index (REI) ≥5 events/h was observed in 6/16 (37.5%) and 8/15 (53.3%), respectively. **Conclusions:** This pilot provides feasibility, data completeness, and variability estimates for a larger controlled cohort. The small uncontrolled sample, non-standardized laboratory timing, treatment heterogeneity, and missing upstream referral logs prevent obesity-specific, mechanistic, or clinically actionable conclusions.

## 1. Introduction

Over recent decades, obesity prevalence has increased substantially, making obesity a major public health concern. Worldwide adult obesity prevalence more than doubled between 1990 and 2022, and the World Health Organization estimates that approximately 16% of adults were living with obesity in 2022 [1,2]. In Spain, the 2023 national health survey estimated that 15.2% of adults aged 18 years or older were living with obesity [3]. In the WHO European Region, overweight and obesity combined affect almost 60% of adults [4]. These figures support structured preconception, antenatal, and postpartum counseling and management for women with obesity [5].

Maternal obesity during pregnancy has important implications for health, pregnancy outcomes, and cardiometabolic risk in both the mother and offspring [6]. It is associated with a higher risk of gestational diabetes mellitus (GDM), hypertensive disorders of pregnancy, cesarean delivery, fetal macrosomia, and other adverse perinatal outcomes [6]. Adverse intrauterine exposures may induce structural, metabolic, and epigenetic changes that increase susceptibility to chronic diseases throughout life [7,8,9]. Within this framework, maternal obesity represents a potentially modifiable exposure, and maternal nutrition contributes to the intrauterine environment.

Sleep disruption is common during pregnancy. Large observational data describe frequent poor sleep quality, insufficient sleep, nocturnal awakenings, insomnia symptoms, excessive daytime sleepiness, and restless-legs symptoms across gestation, while systematic-review evidence indicates that longitudinal sleep trajectories are heterogeneous rather than uniform [10,11]. These non-respiratory manifestations may impair daytime function and coexist with metabolic and obstetric risk factors. However, their longitudinal course and relationship with routinely collected prenatal measures remain incompletely described.

Sleep-disordered breathing, including obstructive sleep apnea (OSA), is also relevant during pregnancy and in women with obesity [12,13,14,15,16,17,18,19,20,21]. Obesity is a major modifiable risk factor for OSA, and the relationship may be self-reinforcing: OSA-related sleep fragmentation, intermittent hypoxia, daytime fatigue, reduced physical activity, and disturbances in appetite and energy-balance regulation may make weight management more difficult [19,20,22]. OSA may nevertheless remain unrecognized during pregnancy. These considerations motivated the study-specific BMI-based referral approach used among women who reached the sleep unit in this pilot. Objective respiratory monitoring was obtained within the broader study; however, respiratory findings are reported descriptively and are not used to support the primary non-respiratory conclusions.

Sleep fragmentation and insomnia may influence appetite regulation, glucose homeostasis, fatigue, and health behaviors [15]. Experimental and review literature indicates that sleep deprivation can alter central appetite–regulatory pathways involving orexin, ghrelin, leptin, and insulin [22], while meta-analytic evidence associates both short and long sleep duration during pregnancy with GDM risk [23]. Suboptimal micronutrient status, particularly iron and folate status, may also contribute to fatigue or restless-legs symptoms [24,25]. These overlapping pathways support an integrated assessment of sleep, nutrition, and routine clinical measures, but a small observational pilot study cannot establish mechanistic or causal relationships.

Repeated assessment at clearly defined gestational stages is important because sleep continuity and hematological measures change throughout pregnancy. In a pilot study, study accrual, retention, questionnaire completion, and availability of repeated clinical data are also relevant outcomes for planning a larger controlled cohort [26].

Appropriate nutritional supplementation during pregnancy is important for preventing maternal and fetal micronutrient deficiencies. Women with obesity may have an increased risk of suboptimal folate, vitamin D, vitamin B12, or iron status [24,25]. In this study, however, the nutritional domain was restricted to prescribed supplement exposure recorded in routine clinical records. It did not assess total dietary intake, actual supplement consumption or adherence, exact nutrient doses or formulations, or serum micronutrient status. Because prescribed preparations frequently contain overlapping micronutrients, exposure was summarized using generic prescription categories rather than brand names alone.

Accordingly, this single-center prospective pilot study aimed to describe the longitudinal evolution of anthropometric measures, prescribed nutritional supplement exposure, non-respiratory sleep symptoms, routine hematological and blood-count-derived inflammatory indices, and obstetric and neonatal outcomes in pregnant women with obesity. A co-primary feasibility aim was to document observed study accrual at the sleep unit and data completeness across study domains. Secondary exploratory analyses examined the direction and imprecision of associations between baseline BMI, interval gestational weight change, sleep scores, and adverse obstetric or neonatal outcomes. The study was not designed to determine whether observed patterns were specific to obesity.

## 2. Materials and Methods

### 2.1. Study Design

A single-center prospective pilot study was conducted at Burgos University Hospital between July 2023 and September 2024. Seventeen adult pregnant women with BMI ≥ 30 kg/m^2^ at the beginning of pregnancy and singleton pregnancies reached the sleep unit and consented to participate. The study was designed to evaluate the feasibility of repeated multidimensional assessment and to obtain preliminary estimates for planning a larger controlled cohort, rather than to test confirmatory hypotheses. Feasibility outcomes were observed in study accrual at the sleep unit, consent among women who attended, availability of paired first- and third-trimester assessments, questionnaire completion, availability of repeated routine laboratory data, and availability of delivery outcomes. The number of women informed or referred in Obstetrics and the number who did not subsequently attend the sleep unit were not prospectively logged and could not be reconstructed reliably; consequently, overall referral uptake and consent among all women initially informed cannot be estimated. Reporting was informed by recommendations for non-randomized pilot and feasibility studies [26].

### 2.2. Participants and Selection Criteria

Pregnant women with first trimester BMI ≥ 30 kg/m^2^ were informed about the study in the first Obstetrics consultation and could be referred to the sleep unit because obesity confers an elevated risk of OSA, rather than because they had sleep symptoms or suspected sleep pathology. This was an exploratory and study-specific referral approach, and not an established routine clinical protocol. Its longer-term purpose was to assess the feasibility of a future Obstetrics-to-Sleep Unit pathway for pregnant women at increased risk of OSA; at the study center, systematic OSA risk assessment and referral of pregnant women with obesity were not part of routine obstetric care. Not all women informed in Obstetrics subsequently attended the sleep unit, but the number informed, referred, or not attending was not recorded. Seventeen women reached the sleep unit and all accepted participation, so consent among sleep unit attendees was 100%, whereas uptake from the initial Obstetrics contact cannot be calculated.

No formal power calculation was performed because the study was not intended to provide confirmatory estimates of association or treatment effect. The achieved sample reflects enrollment during the defined study-accrual period. The revised analysis therefore emphasizes feasibility, data completeness, observed variability, effect estimates, and confidence intervals rather than statistical significance.

The inclusion criteria were age ≥18 years, singleton pregnancy, and BMI ≥ 30 kg/m^2^ in the first trimester of gestation, up to week 12.

The exclusion criteria were multiple pregnancy, a pre-existing diagnosis or clinical suspicion of OSA or another sleep disorder before study entry, previous or current treatment with continuous positive airway pressure (CPAP), psychophysical inability to complete the questionnaires, >50% central apneas or Cheyne–Stokes respiration, regular use of hypnotic or sedative drugs, and severe disease that could affect the study objectives or compromise interpretation. Referral to the sleep unit was based on obesity-related OSA risk and not on sleep symptoms. Consequently, the enrolled cohort comprised women referred because of obesity but without previously recognized or clinically suspected sleep disease. The analytic dataset did not include the operational screening assessment used to define clinical suspicion or the number excluded for this reason. Objective polygraphy subsequently identified previously unrecognized sleep-disordered breathing in some enrolled participants. Exclusion of women with known or suspected sleep disease remains a source of selection bias and limits external validity.

### 2.3. Procedure and Data Collection

Protocol-based research assessments were scheduled in the first trimester (T1; up to 12 gestational weeks) and third trimester (T3). At the initial visit, after written informed consent, sociodemographic, clinical, anthropometric, obstetric, and sleep variables were collected. Median gestational age was 9.6 weeks (IQR 9.0–12.0; *n* = 17) at the T1 visit and 28.9 weeks (IQR 28.4–29.4; *n* = 15) at the T3 visit.

**Figure 1:**
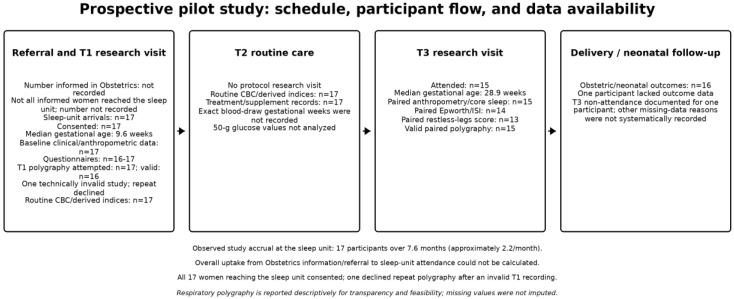
Prospective pilot study schedule, participant flow, and observed data availability. The numbers informed or referred in Obstetrics and not subsequently attending the sleep unit were not recorded. All 17 women who reached the sleep unit consented. Protocol-based visits occurred in T1 and T3; one T1 polygraphy was technically invalid and the participant declined a repeat study. T2 laboratory and treatment data were extracted from routine records. Respiratory polygraphy is reported descriptively for transparency and feasibility but was not used in the primary association analyses.

Routine laboratory values, prescribed nutritional supplements and preventive treatments, and obstetric and neonatal outcomes were extracted from the medical record. There was no protocol-based T2 research visit: T2 laboratory and treatment data reflect routine clinical care. Exact gestational age and clinical indication for individual blood draws were not recorded in the analytic dataset; laboratory values could therefore be classified only by trimester. Figure 1 summarizes the assessment schedule and observed data availability.

### 2.4. Anthropometric Assessment

Maternal weight and height were measured using standardized procedures with a standard calibrated hospital scale and stadiometer, and BMI was calculated as kg/m^2^. BMI categories were defined according to World Health Organization (WHO) criteria as follows: underweight (<18.5 kg/m^2^), normal weight (18.5–24.9 kg/m^2^), overweight (25.0–29.9 kg/m^2^), class I obesity (30.0–34.9 kg/m^2^), class II obesity (35.0–39.9 kg/m^2^), and class III obesity (≥40.0 kg/m^2^) [4].

Neck circumference was measured at the level of the cricothyroid membrane using a non-extensible tape measure. Anthropometric measurements were scheduled in T1 and T3. The change between the two study visits was calculated; because prepregnancy weight and delivery weight were not used, this quantity represents interval change between assessments rather than total gestational weight gain.

### 2.5. Routine Hematological Measures and Derived Blood-Count Indices: Data Extraction and Definitions

Available routine complete blood count parameters obtained during T1, T2, and T3 were extracted from the medical record. These tests were performed as part of clinical care rather than at protocol-mandated research time points.

For hematological evaluation, hemoglobin (g/dL), mean corpuscular volume (MCV, fL), and mean corpuscular hemoglobin (MCH, pg) were collected. Anemia was defined using trimester-specific WHO 2024 thresholds: hemoglobin <11.0 g/dL in T1 and T3 and <10.5 g/dL in T2 [27]. Routine-care blood glucose measurements from standard laboratory testing were available for 16 participants in T1, one participant in T2, and 16 participants in T3. These were routine laboratory glucose values and were not obtained after an oral glucose load. Because exact gestational timing, fasting status, clinical indication, and pre-analytical conditions were not recorded, and data availability was highly unbalanced across trimesters, these values were excluded from the longitudinal biomarker analysis and are not presented as a metabolic trajectory. Clinically diagnosed gestational diabetes mellitus was retained as an obstetric outcome. During the study period, routine GDM screening at Burgos University Hospital followed a two-step paradigm. The 50 g and 1 h O’Sullivan glucose challenge test was considered positive when the 1 h glucose concentration was >140 mg/dL. Women with a positive screening result underwent a fasting 100 g, 3 h oral glucose tolerance test. GDM was diagnosed when at least two glucose values met or exceeded the following thresholds: 105 mg/dL fasting, 190 mg/dL at 1 h, 165 mg/dL at 2 h, and 145 mg/dL at 3 h. GDM was counted only when the diagnosis was documented in the obstetric record; the non-standardized routine glucose values excluded from the longitudinal analysis were not used to retrospectively assign GDM.

For exploratory evaluation of systemic inflammation, absolute neutrophil, lymphocyte, and monocyte counts and platelet counts (all recorded as ×10^3^/µL) were used. NLR was calculated as absolute neutrophils/absolute lymphocytes; PLR as platelets/absolute lymphocytes; MLR as absolute monocytes/absolute lymphocytes; SII as absolute neutrophils × platelets/absolute lymphocytes; and SIRI as absolute neutrophils × absolute monocytes/absolute lymphocytes. NLR, PLR, and MLR are dimensionless; SII and SIRI retain units of ×10^3^/µL when counts are entered in ×10^3^/µL.

These indices were interpreted exclusively as non-specific, indirect markers derived from peripheral blood counts. They do not directly measure decidual or placental immune activity. Intercurrent infection, inflammatory disease activity, corticosteroid exposure, and the clinical indication for each blood draw were not systematically captured and could not be adjusted for; routine-care sampling may therefore introduce indication and timing bias.

### 2.6. Prescribed Nutritional Supplements and Preventive Treatments: Data Extraction and Classification

Prescribed nutritional supplements and preventive treatments were extracted from the medical record. The database contained separate fields for four commercial prenatal multivitamin/mineral preparations, Yodocefol (Italfarmaco S.A., Alcobendas, Madrid, Spain), a generic iodine/folic acid/B-vitamin combination, vitamin D, and therapeutic or preventive treatments. For reporting, the four commercial prenatal products were combined into an ‘any branded prenatal multivitamin/mineral preparation’ category, and Yodocefol or the generic combination were combined into an ‘iodine/folic acid/B-vitamin preparation’ category using participant-level OR coding. Each participant was counted once within each category per trimester; categories were not mutually exclusive because more than one preparation could be prescribed. Accordingly, nutritional assessment was limited to categorical prescription exposure (Yes/No) and should not be interpreted as total dietary intake, actual supplement use, adherence, exact nutrient dose or formulation, or biochemical micronutrient status. Individual nutrient doses, biochemical formulations, adherence logs, and serum ferritin, folate, vitamin B12, and vitamin D concentrations were unavailable.

Supplement and treatment exposures were summarized descriptively by trimester. Because prescriptions were individualized according to clinical risk and laboratory findings, no treatment-effect comparisons were undertaken.

### 2.7. Sleep Assessment

To assess sleep-related symptoms, participants completed standardized questionnaires and clinical questions in the first and third trimesters of pregnancy.

The present analysis included non-respiratory sleep symptoms, mainly sleep duration, nocturnal awakenings, daytime sleepiness, insomnia symptoms, and symptoms compatible with restless-legs syndrome.

Daytime sleepiness was assessed using the Epworth Sleepiness Scale (range 0–24) [28]. For the prespecified database categorization used in this analysis, scores <12 indicated no significant daytime sleepiness and scores ≥12 indicated significant daytime sleepiness; this threshold is reported as a study-specific database categorization rather than as a universal clinical cutoff. Insomnia was assessed using the Insomnia Severity Index (ISI; range 0–28) [29] and categorized as no clinical insomnia (0–7), subclinical insomnia (8–14), moderate clinical insomnia (15–21), or severe insomnia (22–28). Restless-legs symptom severity was recorded on a 0–40 scale using the standard severity bands of the International Restless-Legs Syndrome Study Group rating scale [30]: absent (0), mild (1–10), moderate (11–20), severe (21–30), or very severe (31–40). The original questionnaire form was not retained in the analytic dataset; therefore, the manuscript reports the recorded score and categories without assuming additional instrument details. For descriptive presentation of restless-legs symptom categories, the moderate, severe, and very severe categories were combined because no participant had a score in those ranges. Direct clinical questions concerning nocturnal awakenings, perceived excessive daytime sleepiness, poor nocturnal rest, concentration problems, and related symptoms were analyzed separately from the score-based questionnaire categories. Continuous questionnaire scores and grouped categories were both reported when appropriate.

Within the broader study protocol, participants underwent type III home cardiorespiratory polygraphy using a Nox T3s™ portable monitor (Nox Medical, Reykjavík, Iceland) in T1 and T3. The analytic database contained recording duration and the global apnea–hypopnea index reported by the sleep unit. Because type III polygraphy did not include electroencephalography, objectively measured total sleep time was unavailable; for methodological accuracy, the device-reported index is presented in this manuscript as a recording-time respiratory event index (REI), while acknowledging that it was labeled AHI in the source clinical record [31]. For descriptive categorization, REI < 5 events/h was classified as no OSA, 5 to <15 events/h as mild OSA, 15 to <30 events/h as moderate OSA, and ≥30 events/h as severe OSA. A recording was considered valid when the sleep unit accepted it as technically interpretable for clinical reporting. The analytic dataset did not retain event-by-event scoring rules, minimum recording duration requirements, channel-specific signal-quality criteria, or information on whether automated scoring was manually edited; these details could not be reconstructed retrospectively. All 17 participants attempted the T1 study; one recording was technically invalid and the participant declined a repeat study, leaving 16 valid T1 recordings. The revised report includes technical availability, median REI, and diagnostic categories descriptively. Respiratory variables were not included in the exploratory association analyses and are not used to support the primary non-respiratory conclusions.

### 2.8. Obstetric and Neonatal Outcomes: Data Collection and Definitions

During gestational follow-up, the following obstetric variables were collected: gestational hypertension, GDM, preeclampsia, eclampsia, congenital anomalies, intrauterine growth restriction, altered fetal growth, threatened preterm labor, and miscarriage.

After delivery, intrapartum, postpartum, and neonatal variables were extracted from the medical record. Newborns were classified according to gestational age, birth weight, and birth weight percentile for gestational age.

### 2.9. Exploratory Composite Outcomes

Two composite outcomes were constructed for exploratory purposes.

The adverse obstetric outcome included gestational hypertension, GDM, preeclampsia, eclampsia, threatened preterm labor, miscarriage, or prematurity.

The adverse neonatal outcome included low birth weight, ventilatory support, admission to the neonatal intensive care unit (NICU), perinatal death, or neonatal death.

### 2.10. Statistical Analysis

The analyses were designed to characterize feasibility, data completeness, observed variability, and the magnitude and imprecision of candidate effects for future study planning. They were not designed as confirmatory tests. Primary emphasis was placed on effect estimates with 95% confidence intervals and on clinically interpretable variability; *p*-values were retained only as secondary exploratory descriptors.

Statistical analyses were performed using RStudio version 2026.01.1+403 for macOS (Posit Software, PBC, Boston, MA, USA). Quantitative variables were summarized using the median and interquartile range (IQR), and categorical variables using absolute frequency and percentage. Missing values were not imputed. Each paired or repeated-measures analysis used complete cases for the relevant endpoint and time points; the denominator is reported for every analysis.

For paired T1–T3 quantitative outcomes, the median paired change and IQR were reported together with the Hodges–Lehmann paired-location estimate and a 95% non-parametric bootstrap confidence interval based on 10,000 participant-level resamples; the Wilcoxon signed-rank *p*-value was reported secondarily. Paired dichotomous variables were compared using McNemar’s test when calculable. For T1–T2–T3 biomarkers, the Friedman test was applied only to participants with values at all three time points; Kendall’s W and a 95% bootstrap confidence interval were used as the effect-size measure. Nutritional supplements, preventive treatments, and obstetric and neonatal outcomes were summarized descriptively.

Exploratory quantitative associations were summarized using Spearman’s rho with 95% bootstrap confidence intervals. Quantitative sleep scores were compared by adverse-outcome status using the Mann–Whitney U test and Cliff’s delta with a 95% bootstrap confidence interval. The adverse obstetric and neonatal composites were retained solely as exploratory planning outcomes because their components differ in clinical severity and the number of events was small.

No multivariable models, covariate adjustments, treatment-effect analyses, or corrections for multiplicity were performed. Consequently, all *p*-values and confidence intervals should be interpreted as descriptive and hypothesis-generating rather than as evidence of confirmed associations.

### 2.11. Ethical Considerations

The study was approved by the Research Ethics Committee for Medicines of the Burgos and Soria Health Area on 30 May 2023 (Ref. CEIm 2936) and was conducted in accordance with the principles of the Declaration of Helsinki. All participants received oral and written information about the study objectives and procedures and provided written informed consent before inclusion.

Data were processed confidentially and pseudonymized in accordance with current personal data protection regulations.

## 3. Results

### 3.1. Descriptive Analysis

Seventeen pregnant women reached the sleep unit between 27 July 2023 and 14 March 2024 and all consented to participate. This corresponds to an observed study accrual of approximately 2.2 participants per month over 7.6 months. Because the number informed or referred in Obstetrics and the number who did not attend the sleep unit were not recorded, this value is an accrual rate among enrolled participants and not an estimate of referral uptake or overall consent among all women approached. Baseline data were available for all 17 participants; neck circumference for 15/17 (88.2%); paired T1–T3 anthropometry and core sleep questions for 15/17 (88.2%); paired Epworth and ISI scores for 14/17 (82.4%); paired restless-legs scores for 13/17 (76.5%); valid polygraphy for 16/17 at T1 and 15/17 at T3; and obstetric/neonatal outcomes for 16/17 (94.1%). All 17 participants attempted T1 polygraphy; one recording was technically invalid and that participant declined a repeat study. One participant was explicitly recorded as not attending the T3 visit; another had no T3 or delivery record and the reason was not recorded. One T3 attendee lacked Epworth and ISI scores. Reasons for the two missing neck circumference measurements and other instrument-specific missing values were not recorded. No missing values were imputed. The sample had a median age of 35 years (IQR 30–37), baseline weight 83.4 kg (IQR 77.6–95.4), height 160 cm (IQR 158–163), BMI 32.4 kg/m^2^ (IQR 30.2–36.5), and neck circumference 35 cm (IQR 34.5–37; *n* = 15) (Table 1).

Regarding clinical history, two participants (11.8%) had pre-existing hypertension and one (5.9%) had a history of anxiety. Eight women (47.1%) were nulliparous. Among participants with available previous obstetric history (*n* = 10), six had a history of miscarriage (60%). Previous gestational hypertension, previous GDM, previous prematurity, previous preeclampsia, and previous eclampsia were recorded in two cases each (20%), whereas previous low birth weight was recorded in one case (10%) (Table 2).

### 3.2. Anthropometric Changes

Among the 15 women with paired T1–T3 data, median weight increased from 83.4 kg (IQR 78.6–94.0) to 89.0 kg (IQR 85.4–99.2). The median paired change was 6.0 kg (IQR 1.4–7.9), with a Hodges–Lehmann estimate of 4.98 kg (bootstrap 95% CI 2.40–7.25; Wilcoxon *p* = 0.004). Median BMI increased from 31.9 kg/m^2^ (IQR 30.2–36.6) to 33.9 kg/m^2^ (IQR 32.7–38.8). The median paired change was 2.3 kg/m^2^ (IQR 0.5–3.1), with a Hodges–Lehmann estimate of 1.95 kg/m^2^ (bootstrap 95% CI 0.91–3.00; *p* = 0.005). These values represent change between study visits rather than total gestational weight gain from prepregnancy to delivery.

### 3.3. Routine Hematological Measures and Derived Blood-Count Indices

Routine hematological measures are shown in Table 3. Complete T1–T2–T3 values were available for all 17 participants for hemoglobin, MCV, MCH, cell counts, and derived indices. Median hemoglobin values were 13.3 g/dL (IQR 12.9–13.6), 12.0 g/dL (IQR 11.4–12.5), and 12.0 g/dL (IQR 11.2–13.0), respectively (Kendall’s W = 0.682, bootstrap 95% CI 0.538–0.817; Friedman *p* < 0.001). MCV medians were 89.4, 92.4, and 89.8 fL (W = 0.315, 95% CI 0.107–0.654; *p* = 0.005). MCH variation was smaller and imprecise (W = 0.172, 95% CI 0.048–0.460; *p* = 0.054).

Routine-care blood glucose measurements were not included in the longitudinal analysis because fasting status, exact gestational timing, clinical indication, and measurement conditions were not recorded and comparability across trimesters could not be established. The T1–T3 comparison and its *p*-value were therefore removed. A gestational diabetes diagnosis is reported with the obstetric outcomes.

For the indirect peripheral blood-count indices, NLR and SII were highest in T2 and lower in T3 (NLR: 3.2, 4.1, and 2.7; W = 0.481, 95% CI 0.253–0.758; SII: 805.7, 968.6, and 766.6; W = 0.388, 95% CI 0.135–0.751). PLR also varied across trimesters (W = 0.388, 95% CI 0.166–0.678). These trajectories are descriptive and should not be interpreted as evidence of a specific systemic or placental inflammatory state.

Using trimester-specific WHO thresholds, no cases of anemia were identified in T1 or T2, whereas 3/17 (17.6%) were recorded in T3. No cases of thrombocytopenia (<140 × 10^3^/µL) were observed. Platelet counts >450 × 10^3^/µL occurred in one participant (5.9%) in each trimester. These clinical categories were described without inferential testing because event counts were small.

### 3.4. Analysis of Non-Respiratory Sleep Symptoms

Non-respiratory sleep measures showed no clear longitudinal change between T1 and T3. Sleep duration was 8 h at both time points (*n* = 15), and nocturnal awakenings were reported by 14/15 (93.3%) in T1 and 15/15 (100%) in T3. In the 14 complete Epworth/ISI pairs, median Epworth scores were 6.0 (IQR 5.25–10.25) and 6.5 (IQR 3.25–10.75), while median ISI scores were 9.0 (IQR 7.25–12.0) and 8.0 (IQR 4.75–9.75). The direct clinical question on excessive daytime sleepiness was endorsed by 6/15 (40.0%) in T1 and 4/15 (26.7%) in T3, whereas significant daytime sleepiness defined by an Epworth score ≥12 was present in 2/14 (14.3%) and 3/14 (21.4%), respectively. This distinction is retained because perceived fatigue/sleepiness and threshold-defined propensity to doze are not equivalent. Restless-legs scores were available as 13 complete pairs and were generally low (Table 4).

Objective type III polygraphy was valid for 16/17 participants at T1 and 15/17 at T3; 15 participants had paired valid studies. Median REI was 2.75 events/h (IQR 1.68–9.23) at T1 and 5.20 events/h (IQR 2.30–8.40) at T3. An REI ≥5 events/h was observed in 6/16 (37.5%) at T1, all classified as mild OSA, and in 8/15 (53.3%) at T3, comprising seven mild and one moderate case. Among paired studies, the median REI change was 0.7 events/h (IQR −1.5 to 1.5), with a Hodges–Lehmann estimate of 0.43 events/h (bootstrap 95% CI −1.15 to 1.80; *p* = 0.599). These respiratory findings are reported descriptively and do not alter the primary non-respiratory focus.

### 3.5. Prescribed Nutritional Supplements and Preventive Treatments

Prescribed supplements and preventive treatments are summarized in Table 5. Any of the four recorded commercial prenatal multivitamin/mineral preparations was prescribed to six women (35.3%) in T1, 7 (41.2%) in T2, and eight (47.1%) in T3. Yodocefol or the separately recorded iodine/folic acid/B-vitamin combination was prescribed to 13 women (76.5%) in T1 and 10 (58.8%) in both T2 and T3. Vitamin D was recorded in eight women (47.1%) in T1 and seven (41.2%) in T2 and T3. Participants were counted once within each category per trimester; categories were non-mutually exclusive because concurrent preparations were possible.

Acetylsalicylic acid was prescribed to seven women (41.2%) in each trimester. Oral iron therapy increased from zero in T1 to two (11.8%) in T2 and four (23.5%) in T3. These treatments were prescribed according to individual clinical indications; their frequencies are descriptive and cannot be interpreted as evidence of treatment effect.

### 3.6. Obstetric and Neonatal Outcomes (n = 16)

Obstetric and neonatal outcomes were available for 16 participants (Table 6). Preeclampsia occurred in 3/16 (18.8%), GDM in 2/16 (12.5%), and gestational hypertension in 1/16 (6.2%). Median gestational age at delivery was 39.4 weeks (IQR 38.4–40.1); 15/16 newborns were born at term and 15/16 had normal birth weight. Two newborns were admitted to the NICU and one required ventilatory support. Given the small denominator and absence of a comparison group, these frequencies should not be interpreted as obesity-specific risk estimates.

### 3.7. Exploratory Associations

Exploratory association analyses were retained only to provide preliminary effect-direction estimates for future study planning. Confidence intervals are emphasized because the small sample produces substantial imprecision.

Baseline BMI showed Spearman correlations of rho = −0.050 with the T1 Epworth score (bootstrap 95% CI −0.495 to 0.367) and rho = −0.394 with the T1 ISI score (95% CI −0.750 to 0.074). Interval weight change showed correlations of rho = 0.396 with the T3 Epworth score (95% CI −0.171 to 0.820) and rho = 0.301 with the T3 ISI score (95% CI −0.277 to 0.715). For the adverse obstetric composite, Cliff’s delta was 0.00 (95% CI −0.64 to 0.67) for T3 Epworth and −0.09 (95% CI −0.73 to 0.56) for T3 ISI. For the adverse neonatal composite, corresponding deltas were −0.08 (95% CI −0.92 to 0.83) and 0.08 (95% CI −1.00 to 1.00). All intervals were wide and compatible with effects in either direction.

## 4. Discussion

This single-center prospective pilot study describes the feasibility and preliminary findings of integrating repeated anthropometric assessment, non-respiratory sleep questionnaires, prescribed nutritional supplement exposure, routine hematological data, and pregnancy outcomes in women with obesity. The study was not powered to identify clinically meaningful associations, and the lack of a normal-weight comparison group prevents attribution of the observed trajectories to obesity rather than to pregnancy physiology.

The principal contribution is operational rather than mechanistic or clinically actionable. Study accrual at the sleep unit averaged approximately 2.2 participants per month during the observed 7.6-month enrollment window, and all 17 women who reached the unit consented. However, the number informed or referred in Obstetrics and the number who did not attend the sleep unit were unavailable, so overall pathway uptake and acceptability cannot be estimated. Paired data availability ranged from 76.5% to 94.1% across the principal domains. These findings identify both the practicality of assessment once women reach the sleep unit and the need for prospective referral and non-attendance logs, standardized laboratory windows, stronger retention procedures, and instrument-specific completion monitoring in a future controlled cohort.

The median interval weight change between the T1 and T3 visits was 6.0 kg. This value should not be compared directly with the recommended 5–9 kg total gestational weight gain for women with prepregnancy BMI ≥ 30 kg/m^2^ [32], because the present measure did not include prepregnancy-to-delivery change. The development and implementation of gestational weight gain standards remain active areas of discussion [33], and recent reviews summarize dietary guidance, physical activity, and emerging telehealth approaches as potential strategies for preventing excessive gestational weight gain [34]. The observed IQR may nevertheless inform the expected variability of a similarly timed endpoint in a future study.

Hemoglobin decreased across pregnancy, and three cases of anemia were identified in T3 using trimester-specific WHO thresholds. Prescribed oral iron increased from 0% in T1 to 23.5% in T3. These observations are compatible with increasing gestational iron requirements, but ferritin, transferrin saturation, dietary intake, adherence, and treatment indication were not available; therefore, iron deficiency and treatment response cannot be inferred [25,35,36].

The modest rise in MCV from T1 to T2, followed by a return toward baseline in T3 should not be attributed to folate or vitamin B12 supplementation. The observed pattern may reflect physiological- or iron-related changes during pregnancy, but ferritin, folate, vitamin B12, reticulocyte, adherence, and treatment-indication data were unavailable. Mechanistic interpretation is therefore not possible.

NLR and SII were highest in T2 and lower in T3. This trajectory is not consistent with the simplified model of a relatively tolerant mid-pregnancy phase bracketed by pro-inflammatory early and late pregnancy states. Pregnancy involves dynamic, cell- and tissue-specific adaptations of innate and adaptive immunity that are not adequately represented by a single peripheral inflammatory trajectory [37]. Peripheral blood-count ratios are non-specific and do not directly represent immune activity at the maternal–fetal interface. The observed T2 peak may reflect normal leukocyte dynamics, differences in exact gestational timing, clinical indications for routine blood sampling, intercurrent illness, medication exposure, or random variation in a small complete-case sample.

Although NLR, PLR, and SII have been investigated as prognostic markers of hypertensive disorders [38], their clinical reliability in this study is limited by the small sample, multiple comparisons, obesity-related low-grade inflammation, and the inability to adjust for infection, corticosteroids, or other inflammatory conditions [39]. Accordingly, these indices should be viewed only as candidate measures whose acquisition and variability can inform a larger standardized study.

Sleep duration and questionnaire scores did not show a clear change between T1 and T3, whereas nocturnal awakenings were reported by almost all participants. A recent systematic review found heterogeneous trajectories of sleep quality, duration, efficiency, timing, and insomnia symptoms across the perinatal period, supporting the view that sleep does not follow a single uniform course during pregnancy [11]. Without a normal-weight pregnant comparison group, the frequency observed in our cohort cannot be interpreted as obesity-specific; frequent awakening is also common during pregnancy. The finding nevertheless supports retaining sleep continuity as a candidate endpoint and using more detailed frequency or actigraphy measures in future work.

The direct question on excessive daytime sleepiness was endorsed by 40.0% in T1, a clinically relevant signal in the context of early-pregnancy fatigue, whereas only 14.3% had an Epworth score ≥12 at that time. This discrepancy may reflect differences between general fatigue or perceived sleepiness and propensity to doze in the situations captured by the Epworth scale. It should not be interpreted as a validated prevalence estimate in women with obesity.

Median Epworth scores remained below the prespecified ≥12 cutoff, but the wide T3 IQR indicates heterogeneity. Future studies should distinguish fatigue from sleepiness, collect work and caregiving schedules, and examine objective respiratory and sleep-continuity measures alongside questionnaires.

The lower median ISI score in T3 was imprecise and could reflect individual variability, adaptation, regression to the mean, or measurement noise. The pilot data do not establish an improvement in insomnia across pregnancy.

Restless-legs symptoms were infrequent. Low ferritin and folate status have been associated with restless-legs symptoms during pregnancy [40]. However, ferritin and folate concentrations were not measured, supplementation was non-randomized, and therapeutic iron was prescribed to only a minority in T3. The low observed frequency therefore cannot be attributed to supplementation.

The heterogeneous use of acetylsalicylic acid, progesterone, heparin, insulin, and supplements reflects individualized risk-based prenatal care. In particular, aspirin prescription is subject to confounding by indication: women at higher baseline risk are more likely to receive treatment. The study neither randomized treatment nor adjusted for indication and therefore cannot estimate the independent effects of these prescriptions on obstetric or neonatal outcomes. Evidence from randomized trials and an observational study supports aspirin use in appropriately selected high-risk women [41,42,43], but the present frequencies are descriptive only.

Similarly, vitamin D and oral iron prescriptions should be interpreted as treatment exposure recorded during routine care rather than as explanatory variables. Baseline nutrient concentrations, dose, adherence, duration, and treatment response were not consistently available, preventing any causal inference about supplementation and outcomes [36,44].

Three participants developed preeclampsia and two developed GDM among the 16 with outcome data. Established meta-analytic evidence links higher prepregnancy BMI with increased risks of preeclampsia [45] and GDM [46]; however, the present proportions are highly imprecise, and this uncontrolled pilot cannot estimate those associations. Comparison with external prevalence estimates is also vulnerable to selection and treatment differences. The favorable majority of neonatal outcomes is descriptive and does not demonstrate protection or treatment benefit.

The pilot results are most useful for planning a future multicenter prospective cohort with a normal-weight comparison group, standardized gestational windows, prespecified primary endpoints, formal screening and refusal logs, complete ascertainment of inflammatory- and medication-related confounders, and participant-level effect estimates with confidence intervals. The descriptive polygraphy results also show that previously unrecognized sleep-disordered breathing can be detected among pregnant women referred because of obesity despite excluding known or suspected disease. These findings provide preliminary feasibility information for a future risk-based referral pathway between Obstetrics and the Sleep Unit, but do not establish the effectiveness or clinical utility of such a pathway.

These data do not yet justify implementing a systematic clinical screening or referral strategy. They support further evaluation of whether a BMI- and risk-based pathway from Obstetrics to the Sleep Unit, together with integrated assessment of sleep, nutrition, and routine clinical measures, is feasible, acceptable, diagnostically useful, and clinically beneficial in a larger controlled study.

### 4.1. Strengths and Limitations

This study has several strengths, including its prospective longitudinal design, the assessment of participants at two clinically relevant stages of pregnancy, and the integration of anthropometric, nutritional, analytical, sleep-related, obstetric, and neonatal variables within the same cohort. The use of standardized sleep questionnaires and symptom scales, together with the availability of routine clinical biomarkers, supports the feasibility of incorporating this multidimensional assessment into prenatal care.

Several limitations are central to interpretation. First, the sample of 17 was too small for stable association estimates, multivariable adjustment, subgroup analysis, or evaluation of treatment effects; the large number of variables further increases the risk of chance findings and type II error. Second, no normal-weight pregnancy comparison group was enrolled, so no trajectory can be attributed specifically to obesity. Third, although referral to the sleep unit was based on first trimester BMI ≥ 30 kg/m^2^ rather than suspected sleep disease, the number of women informed or referred in Obstetrics and the number who did not subsequently attend the sleep unit were not recorded. All 17 women reaching the unit consented, but this cannot be interpreted as 100% acceptability among all women approached. The operational assessment of clinical suspicion and the number excluded for suspected sleep disease were also unavailable. Exclusion of known or suspected OSA may have removed a high-risk subgroup, while objective polygraphy nevertheless identified previously unrecognized OSA among participants. One participant declined repeat polygraphy after a technically invalid T1 recording. Detailed event-scoring rules and technical-validity criteria for the polygraphy reports were not retained in the analytic dataset. Fourth, T2 laboratory data and all routine blood tests were record-extracted rather than protocol-timed; exact gestational ages and clinical indications for blood draws were unavailable. Fifth, inflammatory indices could not be adjusted for infection, corticosteroid exposure, or other inflammatory conditions. Sixth, nutritional assessment was restricted to categorical prescription status (Yes/No) in routine records; individual nutrient doses and formulations, adherence logs, total dietary intake, treatment indications, and serum ferritin, folate, vitamin B12, and vitamin D concentrations were unavailable. Other treatments were heterogeneous and subject to confounding by indication. Seventh, the study did not include lipids, standardized fasting glucose, HbA1c, insulin, or longitudinal blood-pressure trajectories. Eighth, reasons for several individual missing measurements were not systematically recorded. These limitations restrict the study to descriptive feasibility and planning inferences.

### 4.2. Research Implications and Future Study Design

A future study should include a contemporaneous normal-weight comparison group; standardize T1, T2, and T3 research visits and blood-draw windows; record exact gestational age, indication, infection, and corticosteroid exposure; prespecify one or two primary endpoints; and collect the nutrient and metabolic measures required for mechanistic interpretation. The future referral pathway should prospectively define the BMI and clinical-risk algorithm and document each stage: women informed in Obstetrics, women referred, women attending the sleep unit, women consenting, valid and repeated sleep studies, and women diagnosed with OSA. This would permit estimation of referral uptake, non-attendance, acceptability, diagnostic yield, and downstream clinical consequences. It should also consider actigraphy-derived sleep regularity, circadian timing, and meal timing. Observational research has linked maternal sleep variability and circadian misalignment during pregnancy with neonatal structural brain differences [47]. More broadly, circadian systems regulate glucose metabolism and insulin sensitivity [48,49], while the timing of food intake can entrain peripheral metabolic rhythms and influence metabolic health [50]. Accrual and completion estimates from Table 7 can inform the number of sites, recruitment duration, and over-recruitment allowance, but not the expected uptake from Obstetrics until upstream denominators are collected. Candidate endpoint variability and effect estimates from Table 8 should be used cautiously for sample-size planning and validated in a larger external pilot. The potential role of specialized nursing in implementing this pathway should be tested prospectively rather than inferred from the present descriptive data.

## 5. Conclusions

In this single-center prospective pilot cohort of pregnant women with obesity, all 17 women who reached the sleep unit consented, and repeated anthropometric and sleep assessments were feasible, with domain-specific paired data availability of 76.5–94.1%. However, uptake from Obstetrics could not be estimated because the numbers informed, referred, and not attending the sleep unit were not recorded. Weight and BMI increased between T1 and T3, hemoglobin decreased, and peripheral blood-count-derived indices varied across routine trimester measurements. Frequent nocturnal awakenings and subclinical insomnia were observed, but non-respiratory sleep scores showed no clear longitudinal change. Descriptive polygraphy identified an REI ≥ 5 events/h in 37.5% at T1 and 53.3% at T3, indicating that previously unrecognized sleep-disordered breathing was present despite exclusion of known or suspected disease. The small sample, non-standardized record-extracted laboratory timing, treatment heterogeneity, missing data, unavailable upstream referral logs, and absence of a normal-weight comparison group prevent obesity-specific, mechanistic, or clinically actionable conclusions. These findings should be used primarily to design a larger controlled cohort with prespecified feasibility targets, standardized measurements, and effect estimates reported with confidence intervals.

## Figures and Tables

**Table 1:** Baseline clinical, anthropometric, and obstetric characteristics of the included pregnant women.

Variable	*n*	Median (IQR) or *n* (%)
Age, years	17	35 (30–37)
Weight, kg	17	83.4 (77.6–95.4)
Height, cm	17	160 (158–163)
BMI, kg/m^2^	17	32.4 (30.2–36.5)
Neck circumference, cm	15	35 (34.5–37)
Pre-existing hypertension	17	2 (11.8%)
Pre-existing diabetes	17	0 (0%)
Anxiety	17	1 (5.9%)
Depression	17	0 (0%)
Pharmacological treatment for sleep	17	0 (0%)
Alcohol consumption	17	0 (0%)
Smoking	17	0 (0%)
Nulliparity	17	8 (47.1%)
Number of previous pregnancies	17	1 (0–1)
Obesity class I (BMI 30.0–34.9 kg/m^2^)	17	11 (64.7%)
Obesity class II (BMI 35.0–39.9 kg/m^2^)	17	5 (29.4%)
Obesity class III (BMI ≥ 40.0 kg/m^2^)	17	1 (5.9%)
Gestational age at T1 research visit, weeks	17	9.6 (9.0–12.0)
Gestational age at T3 research visit, weeks	15	28.9 (28.4–29.4)

Note: Values are presented as median (IQR) or absolute frequency (%). The *n* corresponds to valid data for each variable. Gestational ages are expressed as decimal weeks after conversion from weeks + days.

**Table 2:** Previous obstetric history among pregnant women with available information (*n* = 10).

Variable	*n* (%)
History of miscarriage	6 (60%)
Previous gestational hypertension	2 (20%)
Previous gestational diabetes mellitus	2 (20%)
Previous prematurity	2 (20%)
Previous low birth weight	1 (10%)
Previous preeclampsia	2 (20%)
Previous eclampsia	2 (20%)

Note: Values are presented as absolute frequency (%).

**Table 3:** Routine hematological measures and blood-count-derived inflammatory indices during pregnancy.

Variable	T1	T2	T3	*p*-Value
Hemoglobin, g/dL (*n* = 17)	13.3 (12.9–13.6)	12 (11.4–12.5)	12 (11.2–13)	<0.001
MCV, fL (*n* = 17)	89.4 (88.4–91.1)	92.4 (90.1–94.3)	89.8 (87.3–92.6)	0.005
MCH, pg (*n* = 17)	29.9 (29.6–30.6)	30.7 (29.6–31.5)	30.3 (28.8–31.4)	0.054
Neutrophils, % (*n* = 17)	70.5 (67.4–74.1)	77.2 (70.2–77.7)	69.1 (65.4–72.9)	0.004
Lymphocytes, % (*n* = 17)	22.6 (21.5–25.1)	18.4 (17.2–22.9)	24.7 (21.7–27.9)	0.001
Monocytes, % (*n* = 17)	3.8 (3.3–4.6)	3.5 (3.1–4)	4.2 (3.6–4.8)	0.094
Platelets, ×10^3^/µL (*n* = 17)	267 (235–303)	243 (214–275)	248 (226–275)	0.662
NLR (*n* = 17)	3.2 (2.6–3.5)	4.1 (3–4.5)	2.7 (2.3–3.5)	<0.001
PLR (*n* = 17)	142.2 (122.2–175)	161.5 (112.6–235.5)	116.3 (96.5–137.8)	0.001
MLR (*n* = 17)	0.2 (0.1–0.2)	0.2 (0.1–0.2)	0.2 (0.1–0.2)	0.405
SII, ×10^3^/µL (*n* = 17)	805.7 (664.6–1052.1)	968.6 (729–1337.5)	766.6 (551.1–872.8)	0.001
SIRI, ×10^3^/µL (*n* = 17)	1 (0.7–1.2)	1.3 (0.9–1.7)	1.1 (0.8–1.5)	0.080

Note: Values are median (IQR). All Friedman analyses shown used the 17 participants with complete T1–T2–T3 values. Kendall’s W estimates and bootstrap 95% confidence intervals for principal candidate endpoints are summarized later as planning parameters for a future controlled cohort. The non-standardized routine-care glucose comparison was removed because fasting status, exact timing, and comparability across trimesters could not be established.

**Table 4:** Non-respiratory sleep symptoms in T1 and T3.

Variable	*n*	T1	T3	*p*
Sleep hours per day, h	15	8 (7–8)	8 (7–8.5)	0.433
Nocturnal awakenings	15	14 (93.3%)	15 (100%)	0.480
Nap	15	9 (60.0%)	7 (46.7%)	1.000
Medication to induce sleep	15	0 (0%)	0 (0%)	—
Leg movements during sleep	15	3 (20.0%)	2 (13.3%)	1.000
Excessive daytime sleepiness	15	6 (40.0%)	4 (26.7%)	1.000
Sleepiness in passive situations	15	5 (33.3%)	8 (53.3%)	0.371
Sleepiness in active situations	15	2 (13.3%)	2 (13.3%)	—
Sleepiness while driving	15	2 (13.3%)	3 (20.0%)	1.000
Feeling of poor night-time rest	14	8 (57.1%)	8 (57.1%)	1.000
Concentration/mental agility problems	14	5 (35.7%)	2 (14.3%)	0.683
Epworth Sleepiness Scale	14	6 (5.25–10.25)	6.5 (3.25–10.75)	0.316
Epworth category (<12 vs. ≥12)	14	No significant daytime sleepiness (<12): 12 (85.7%); Significant daytime sleepiness (≥12): 2 (14.3%)	No significant daytime sleepiness (<12): 11 (78.6%); Significant daytime sleepiness (≥12): 3 (21.4%)	—
Insomnia Severity Index	14	9 (7.25–12)	8 (4.75–9.75)	0.090
Insomnia Severity Index category	14	No clinical insomnia: 4 (28.6%); subclinical insomnia: 10 (71.4%); moderate clinical insomnia: 0 (0%)	No clinical insomnia: 6 (42.9%); subclinical insomnia: 7 (50.0%); moderate clinical insomnia: 1 (7.1%)	—
Restless-legs symptom severity score (0–40)	13	0 (0–0)	0 (0–0)	0.586
Restless-legs symptom severity category	13	No symptoms: 11 (84.6%); mild symptoms: 2 (15.4%); moderate-to-very-severe symptoms: 0 (0%)	No symptoms: 10 (76.9%); mild symptoms: 3 (23.1%); moderate-to-very-severe symptoms: 0 (0%)	—

Note: Values are presented as median (IQR) or absolute frequency (%). Paired quantitative variables were compared using the Wilcoxon test, and dichotomous variables using McNemar’s test when sufficient variability was available. The symbol ‘—‘ indicates that the test was not calculable.

**Table 5:** Nutritional supplements and preventive treatments prescribed during pregnancy.

Supplement or Treatment	T1	T2	T3
Commercial prenatal multivitamin/mineral preparation †	6 (35.3%)	7 (41.2%)	8 (47.1%)
Iodine/folic acid/B-vitamin preparation ‡	13 (76.5%)	10 (58.8%)	10 (58.8%)
Vitamin D	8 (47.1%)	7 (41.2%)	7 (41.2%)
Acetylsalicylic acid	7 (41.2%)	7 (41.2%)	7 (41.2%)
Progesterone	2 (11.8%)	1 (5.9%)	1 (5.9%)
Low-molecular-weight heparin	1 (5.9%)	1 (5.9%)	0 (0%)
Oral iron therapy	0 (0%)	2 (11.8%)	4 (23.5%)
Antiemetic	2 (11.8%)	2 (11.8%)	5 (29.4%)
Insulin	0 (0%)	0 (0%)	1 (5.9%)

Note: Values are *n* (%). † Commercial prenatal multivitamin/mineral preparation combines Exelvit Esencial, Seidibion Prime, Natalben Supra, and Ginecomplex Plus using participant-level OR coding. ‡ Iodine/folic acid/B-vitamin preparation combines Yodocefol and the separately recorded generic combination. Each participant was counted once within each category per trimester; categories are not mutually exclusive. Exact formulations, doses, adherence, and treatment indications were not available. Therapeutic oral iron refers to a separate prescription. These variables therefore represent prescription exposure rather than confirmed intake or micronutrient status.

**Table 6:** Obstetric and neonatal outcomes.

Variable	Median (IQR) or *n* (%)
Gestational hypertension	1 (6.2%)
Gestational diabetes mellitus	2 (12.5%)
Preeclampsia	3 (18.8%)
Eclampsia	0 (0%)
Threatened preterm labor	0 (0%)
Miscarriage	0 (0%)
Gestational age at delivery, weeks	39.4 (38.4–40.1)
Classification by gestational age	Term: 15 (93.8%); preterm: 1 (6.2%)
Term pregnancy	15 (93.8%)
Prematurity	1 (6.2%)
Classification by birth weight	Low birth weight: 1 (6.2%); normal weight: 15 (93.8%)
Low birth weight	1 (6.2%)
Classification by percentile	AGA: 13 (81.2%); LGA: 1 (6.2%); SGA: 2 (12.5%)
Apgar score at 1 min *	9 (8–9)
Apgar score at 5 min **	10 (9.2–10)
Neonatal ventilatory support	1 (6.2%)
NICU admission	2 (12.5%)
Perinatal death	0 (0%)
Neonatal death	0 (0%)
Length of hospital stay, days	4 (3.8–5)

Note: Gestational age at delivery is expressed as decimal weeks after conversion from the weeks + days format. Neonatal classification variables are presented as absolute frequency and percentage. * *n* = 15; ** *n* = 14. AGA: appropriate for gestational age; LGA: large for gestational age; SGA: small for gestational age; NICU: neonatal intensive care unit.

**Table 7:** Feasibility and data-completeness metrics.

Metric	Observed Numerator/Denominator	Observed Rate	Interpretation for Future Study
Study accrual at the sleep unit	17 participants/7.6 months	Approximately 2.2 participants/month	Does not estimate uptake from Obstetrics because the upstream denominator was unavailable.
Consent among women reaching the sleep unit	17/17	100%	All attendees consented; acceptability among all women informed cannot be estimated.
Paired T1–T3 anthropometry	15/17	88.2%	Plan retention procedures and over-recruitment.
Paired core sleep questions	15/17	88.2%	Questionnaire completion was generally high.
Paired Epworth and ISI scores	14/17	82.4%	Allow for instrument-specific missingness.
Paired restless-legs symptom score	13/17	76.5%	This instrument had the lowest completion.
Obstetric/neonatal outcomes	16/17	94.1%	Delivery-record linkage was high but incomplete.
Type III polygraphy availability	T1 attempted 17/17, valid 16/17; T3 valid 15/17; paired valid 15/17	T1 valid 94.1%; T3/paired 88.2%	One T1 recording was invalid and the participant declined a repeat; plan for technical failure and repeat-test refusal.
Complete T1–T2–T3 CBC and derived indices	17/17	100%	Exact blood-draw weeks and indications should be standardized prospectively.
Baseline enrollment data	17/17	100%	Baseline data collection was achievable among enrolled participants.

Note: The numbers informed or referred in Obstetrics and the number not subsequently attending the sleep unit were not prospectively recorded and therefore cannot be reported. All 17 women who reached the sleep unit consented. The study-accrual rate was calculated from the first and last recorded T1 inclusion dates and should not be interpreted as referral uptake from Obstetrics. One T1 polygraphy was technically invalid and the participant declined a repeat study. Missing values were not imputed.

**Table 8:** Preliminary candidate endpoint estimates for planning a future controlled cohort.

Candidate Endpoint	Complete-Case *n*	Observed Estimate	Variability	Effect Estimate (95% CI)
T1–T3 weight	15	T1: 83.4 kg (78.6–94.0); T3: 89.0 kg (85.4–99.2)	SD paired change 4.43 kg	HL 4.98 kg (95% CI 2.40–7.25)
T1–T3 BMI	15	T1: 31.9 kg/m^2^ (30.2–36.6); T3: 33.9 kg/m^2^ (32.7–38.8)	SD paired change 1.80 kg/m^2^	HL 1.95 kg/m^2^ (95% CI 0.91–3.00)
T1–T3 sleep duration	15	T1: 8 h (7–8); T3: 8 h (7–8.5)	SD paired change 1.39 h	HL 0.25 h (95% CI −0.25 to 1.00)
T1–T3 Epworth score	14	T1: 6 (5.25–10.25); T3: 6.5 (3.25–10.75)	SD paired change 2.95	HL 0.50 (95% CI −0.50 to 2.00)
T1–T3 ISI score	14	T1: 9 (7.25–12); T3: 8 (4.75–9.75)	SD paired change 2.93	HL −1.50 (95% CI −3.00 to 0.00)
Hemoglobin T1–T2–T3	17	T1: 13.3 g/dL; T2: 12.0 g/dL; T3: 12.0 g/dL	SD T3–T1 change 1.45 g/dL	Kendall W 0.682 (95% CI 0.538–0.817)
NLR T1–T2–T3	17	T1: 3.2; T2: 4.1; T3: 2.7	SD T3–T1 change 1.15	Kendall W 0.481 (95% CI 0.253–0.758)
SII T1–T2–T3	17	T1: 805.7 × 10^3^/µL; T2: 968.6 × 10^3^/µL; T3: 766.6 × 10^3^/µL	SD T3–T1 change 328.25 × 10^3^/µL	Kendall W 0.388 (95% CI 0.135–0.751)
MCV T1–T2–T3	17	T1: 89.4 fL; T2: 92.4 fL; T3: 89.8 fL	SD T3–T1 change 4.14 fL	Kendall W 0.315 (95% CI 0.107–0.654)
PLR T1–T2–T3	17	T1: 142.2; T2: 161.5; T3: 116.3	SD T3–T1 change 55.34	Kendall W 0.388 (95% CI 0.166–0.678)

Note: Hodges–Lehmann and Kendall’s W confidence intervals were generated by non-parametric participant-level bootstrap resampling. SD of paired change is provided as a planning parameter; a future sample-size calculation will also require a prespecified primary endpoint and clinically meaningful target difference.

## Data Availability

The data presented in this study are available on request from the corresponding author due to privacy and ethical restrictions.

## Abbreviations

AGA, appropriate for gestational age; BMI, body mass index; CPAP, continuous positive airway pressure; GDM, gestational diabetes mellitus; IQR, interquartile range; ISI, Insomnia Severity Index; LGA, large for gestational age; MCH, mean corpuscular hemoglobin; MCV, mean corpuscular volume; MLR, monocyte-to-lymphocyte ratio; NICU, neonatal intensive care unit; NLR, neutrophil-to-lymphocyte ratio; OSA, obstructive sleep apnea; PLR, platelet-to-lymphocyte ratio; REI, respiratory event index; SGA, small for gestational age; SII, systemic immune-inflammation index; SIRI, systemic inflammatory response index; T1, first trimester; T2, second trimester; T3, third trimester; WHO, World Health Organization.
